# Parental asthma and risk of offspring asthma from childhood to adolescence: a population-based cohort study

**DOI:** 10.1136/bmjresp-2025-003608

**Published:** 2026-01-20

**Authors:** Marianne Rørholt Grefslie, Siri Eldevik Håberg, Maria C Magnus, Tone K Omsland, Per Magnus

**Affiliations:** 1 Centre for Fertility and Health, Norwegian Institute of Public Health, Oslo, Norway; 2 Department of Paediatric Medicine, Oslo University Hospital, Oslo, Norway; 3 Department of Global Public Health and Primary Care, University of Bergen, Bergen, Norway; 4 Department of Community Medicine and Global Health, University of Oslo, Oslo, Norway

**Keywords:** Asthma Epidemiology, Paediatric asthma, Asthma, Asthma Mechanisms, Clinical Epidemiology

## Abstract

**Background:**

Maternal asthma has been associated with a higher risk of asthma in early childhood compared with paternal asthma, but it is unclear whether this difference persists into adolescence.

**Methods:**

We analysed 55 643 children from the Norwegian Mother, Father and Child Cohort Study. Parental asthma was self-reported during pregnancy; offspring asthma was reported by mothers at ages 3, 7 and 14 years. Logistic regression models estimated associations between parental asthma and offspring asthma at each age, adjusting for maternal age, parental prepregnancy body mass index, parental education, parental smoking and parental atopic conditions, including atopic eczema and pollen/hay fever.

**Results:**

Asthma prevalence among offspring was 6.5% at age 3, 5.2% at age 7 and 5.4% at age 14. Compared with children of non-asthmatic parents, adjusted ORs for asthma at age 3 were 3.11 (95% CI 2.73 to 3.54) for maternal asthma only and 2.25 (95% CI 1.97 to 2.56) for paternal asthma only. Similar patterns were observed at ages 7 (maternal OR 2.96 (95% CI 2.54 to 3.45); paternal OR 2.36 (95% CI 2.03 to 2.75)) and 14 (maternal OR 3.03 (95% CI 2.46 to 3.73); paternal OR 1.95 (95% CI 1.57 to 2.43)). At age 3, maternal asthma was associated with higher odds in boys (OR 3.30) than girls (OR 2.82), and higher absolute risk (19.2% vs 12.2%). However, interaction tests by offspring sex were not statistically significant.

**Conclusions:**

Maternal asthma conferred a consistently stronger risk of offspring asthma than paternal asthma, from early childhood into adolescence. This effect appeared slightly stronger in boys in early childhood, though sex differences were not statistically significant.

**WHAT IS ALREADY KNOWN ON THIS TOPIC**

- Maternal asthma is found to increase risk of offspring asthma in early childhood more than paternal asthma.

**WHAT THIS STUDY ADDS**

- Our findings indicate that the maternal effect on offspring asthma remains stronger than the paternal effect well into adolescence.

**HOW THIS STUDY MIGHT AFFECT RESEARCH, PRACTICE OR POLICY**

- Maternal asthma history is important in predicting childhood asthma risk, and improving our understanding of these associations may enhance early identification and follow-up of children at increased risk of asthma.

## Introduction

 Asthma is the most common chronic disease in childhood, although prevalence rates vary across studies. In Europe, the prevalence of asthma among children has been reported to range from 2% to 14% at age 4 years, 7% to 12% at age 6–7 years and 4% to 20% in the 13–14 year age group.^1^,^6^ Although challenging to measure, it is assumed that the prevalence of asthma decreases with increasing age, highlighting the dynamic nature of the condition throughout early life.^1^,^6^ Asthma is characterised by reversible airway obstruction and inflammation^7^ and is believed to result from a complex interplay between environmental exposures and an underlying genetic predisposition.^8^,^10^

A maternal effect on offspring asthma has been reported by several researchers.^11^,^14^ Previous studies have shown that a maternal history of asthma has a substantially greater impact on the offspring’s risk during early childhood compared with a paternal history. However, findings become more conflicting after the age of 5 years.^1114^,^16^ While two studies reported similar effects from maternal and paternal asthma after age five,^11 14^ another study in children aged 9–11 years found that paternal asthma had a stronger influence on offspring asthma than maternal asthma.^17^

Estimates for the maternal effect on offspring asthma during the teenage years are limited. The formerly found maternal effect has mainly been shown in small study populations. Since few studies have had data on paternal asthma, direct comparisons of maternal and paternal effects have been lacking.

The main objective of this study was to explore potential differences in the risk of asthma according to maternal versus paternal history of asthma, and if this difference persists until teenage years.

## Materials and methods

### Study population

**Figure 1:**
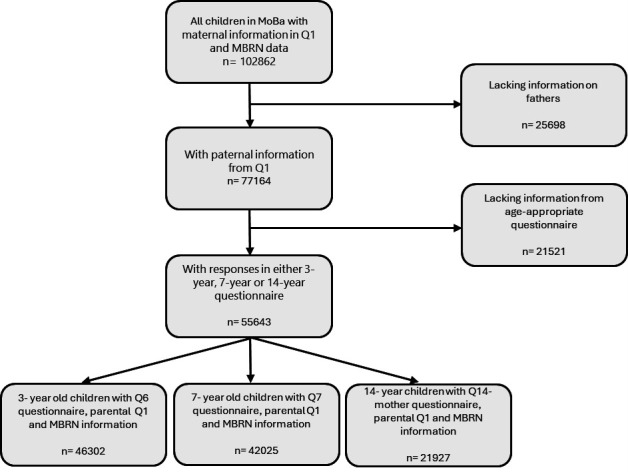
Flowchart showing selection of study participants. Q1 was sent out at recruitment at approximately week 15 of pregnancy to mothers and fathers. Q6 was sent out to mothers when the child was 36 months, Q7 to mothers when the child was 7 years old and Q14-mother to mothers when the child was 14 years old. MBRN, Medical Birth Registry of Norway; MoBa, Norwegian Mother, Father and Child Cohort Study.

We studied participants in the Norwegian Mother, Father and Child Cohort Study (MoBa).^18^ Pregnant women were recruited between 1999 and 2008, at approximately 15–18 weeks of gestation. Participation rate among invited women was 41%. Partners were invited to participate concurrently.^18^ Women could participate with more than one pregnancy, resulting in approximately 95 000 mothers, 75 500 fathers and 114 500 children being recruited.^19^ Participants gave written informed consent. MoBa is regulated by the Norwegian Health Registry Act. MoBa was linked to the Medical Birth Registry of Norway (MBRN)^20^ using unique 11-digit personal identification numbers. Parents completed comprehensive questionnaires during pregnancy and after birth. We included all children with asthma data at 3, 7 or 14 years, and parental data at baseline from questionnaires and MBRN (figure 1). This resulted in a total study population of 55 643 children. Questionnaire response rates varied: 46 302 at age 3 (83.2%), 42 025 at age 7 (75.5%) and 21 927 at age 14 (39.4%) (figure 1).

### Patient and public involvement

Patients and the public were not involved in the design, conduct, reporting or dissemination plans of this research. This study was based on secondary analysis of data from the MoBa, which collects information via questionnaires and national registries. Although participants did not directly contribute to the research questions or methods for this specific analysis, their ongoing engagement in the cohort is vital for enabling population health research. Study findings will be shared through scientific publications and communication channels of the Norwegian Institute of Public Health.

### Maternal and paternal asthma

In Q1, mothers were asked whether they had asthma before or during the pregnancy, while fathers were asked if they ever had asthma and if so, the age of onset. Maternal and paternal asthma was defined as a baseline positive report of asthma by the mother or father respectively, at any point before or during the pregnancy. Information on asthma severity or current disease activity was not available, and age of onset was only collected for the father. As illustrated in figure 1, both parents needed to have completed the parental questionnaire to be included.

### Maternal and paternal atopic traits

Parental atopy was self-reported through separate maternal and paternal questionnaires at recruitment as a history of either atopic eczema or pollen/hay fever, or both, as a current or previous health condition. The term ‘pollen/hay fever’ reflects the questionnaire wording and corresponds to seasonal allergic rhinitis rather than perennial forms.

### Childhood asthma

Childhood asthma was defined based on maternal reports of offspring asthma as a current health problem at ages 3, 7 and 14 years.^18^ Children were considered non-asthmatic if asthma was explicitly denied or if responses to asthma questions were missing but other items were completed. Objective clinical measures (eg, spirometry or physician diagnosis) were not available. However, maternally reported asthma in MoBa has been validated against dispensed asthma medications recorded in the Norwegian Prescription Database (NorPD). Among 2056 mothers, maternal report of antiasthmatic use showed sensitivity of 85.0% and specificity of 96.8%. For maternal report of current asthma verified by a doctor, sensitivity was 80.3%, and only 1.2% of children without reported asthma had dispensed antiasthmatics.^21^

### Childhood atopic traits

Childhood atopic traits were defined as the presence of either atopic eczema or allergy to pollen/hay fever reported by mothers as a current health problem at 3, 7 and 14 years. The term ‘pollen/hay fever’ reflects the questionnaire wording and corresponds to seasonal allergic rhinitis rather than perennial forms.

### Covariates

**Figure 2:**
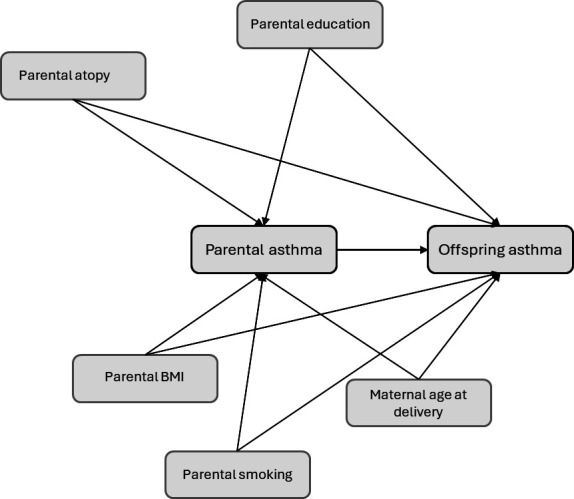
Diagram of confounders. Covariates having the potential to influence both the risk of parental asthma as well as offspring asthma were considered confounders and adjusted for. BMI, body mass index.

Characteristics plausibly associated with both parental asthma and offspring asthma development were included as potential confounders and adjusted for in the analyses (figure 2). These included maternal age at delivery (categorised as <25, 25–29, 30–34, 35–30, 40+), maternal and paternal education (9-year elementary school, 1–3 years of high school, 4+ years of university/college), prepregnancy body mass index (BMI) for both parents (underweight, normal weight, overweight, obese; categorised according to WHO guidelines) and maternal and paternal smoking at recruitment. These were treated as confounders due to their potential to influence both parental asthma status and offspring asthma development.

Maternal and paternal atopy (binary variables) were included as indicators of genetic predisposition, as they increase the risk of asthma in both parent and offspring, hence treated as confounders. However, because atopy may lie partly on the causal pathway between parental asthma and childhood asthma, its inclusion could lead to overadjustment. We therefore interpret adjusted estimates with this limitation in mind. birth weight (categorised as 250–999 g, 1000–1499 g, 1500–2499 g, 2500–4000 g) and delivery by caesarean section (binary, for all causes) are likely mediators, hence not adjusted for. Offspring sex was treated as a potential modifying factor and not adjusted for.

Maternal and paternal characteristics were obtained from parent-reported questionnaires, while sex, birth weight and delivery by caesarean section were acquired from the MBRN.

### Follow-up handling of missing data

Follow-up questionnaires were administered when the child was 3, 7 and 14 years old, and participation declined over time, consistent with patterns observed in long-term cohort studies. For each age, analyses were conducted using a complete-case approach, including all children with available asthma data at that time point. This allowed retention of participants who responded at earlier ages even if they did not provide data at later follow-ups.

Attrition was primarily related to sociodemographic factors such as education and smoking, and patterns were broadly similar for mothers and fathers (online supplemental table 3), reducing the likelihood of differential loss by parental asthma status. Because missingness was largely due to questionnaire non-response rather than incomplete covariate data, we did not apply multiple imputation. To assess potential bias, we compared baseline characteristics of responders at ages 3, 7 and 14 with the full cohort (online supplemental table 3).

### Statistical analysis

We calculated the prevalence of asthma at child ages 3, 7 and 14 years, stratified by parental asthma status: maternal only, paternal only, both parents with asthma or neither parent with asthma.

Logistic regression estimated the crude and adjusted ORs for offspring asthma at each age point, based on parental asthma status, using children with non-asthmatic parents as reference. Adjusted models included maternal age at delivery, maternal and paternal prepregnancy BMI, maternal and paternal education, maternal and paternal smoking, as well as maternal and paternal atopy. To account for the non-independence of observations arising from parents contributing more than one child to the cohort, we used robust SEs clustered at the maternal level in all regression analyses. This approach adjusts for within-family correlation and provides valid estimates of uncertainty when siblings share the same parental exposures.

In addition, stratified analyses were conducted to assess potential effect modification by offspring sex and by presence of atopic traits. Subgroup analyses by child sex and parental atopic traits were considered exploratory and were not adjusted for multiple comparisons. Statistical analyses were conducted with Stata V.18.0 (StataCorp, College Station, Texas, USA).

## Results

The distribution of parental and child characteristics by child asthma status at ages 3, 7 and 14 years is presented in online supplemental table 1. A total of approximately 46 000 mothers provided asthma information for their child at age 3. This number decreased to roughly 42 000 at age 7 and 22 000 at age 14, reflecting gradual attrition due to non-response to later questionnaires rather than withdrawal from the cohort. The prevalence of asthma was 6.5% at 3 years, 5.2% at 7 years and 5.4% at 14 years of age. Asthma was more common among children of obese parents, smoking parents and parents with a lower educational attainment (online supplemental table 1).

### Effect of parental history of asthma at the different age points

At age 3, compared with children with no parental asthma, children with an asthmatic mother only had an OR of 3.42 (95% CI 3.06 to 3.82) for current asthma, while those with an asthmatic father only had an OR of 2.13 (95% CI 1.89 to 2.39) (table 1). The risk was highest among children with both parents affected, with an OR of 5.12 (95% CI 3.93 to 6.66). After adjusting for potential confounders, the maternal effect was slightly attenuated, and the paternal effect slightly increased, but the overall pattern persisted (table 1).

**Table 1:** Crude and adjusted ORs with 95% CIs for asthma at 3, 7 and 14 years according to maternal and paternal asthma status

Parental asthma status		n	Asthma Yes	Absolute risk	Risk difference	Crude OR (95% CI)	Adjusted OR
**Children 3 years**							
Non-asthmatic mothers	Non-asthmatic fathers	39 187	2046	5.20%	0%	1	1
Asthmatic mothers	Non-asthmatic fathers	3060	485	15.90%	10.70%	3.42 (3.06 to 3.82)	3.11 (2.73 to 3.54)
Non-asthmatic mothers	Asthmatic fathers	3705	389	10.50%	5.30%	2.13 (1.89 to 2.39)	2.25 (1.97 to 2.56)
Asthmatic mothers	Asthmatic fathers	350	77	22.00%	16.80%	5.12 (3.93 to 6.66)	4.18 (3.07 to 5.69)
**Children 7 years**							
Non-asthmatic mothers	Non-asthmatic fathers	35 585	1464	4.10%	0%	1	1
Asthmatic mothers	Non-asthmatic fathers	2759	356	12.90%	8.80%	3.45 (3.04 to 3.92)	2.96 (2.54 to 3.45)
Non-asthmatic mothers	Asthmatic fathers	3383	306	9.10%	5%	2.32 (2.04 to 2.64)	2.36 (2.03 to 2.75)
Asthmatic mothers	Asthmatic fathers	298	62	20.80%	16.70%	6.15 (4.59 to 8.23)	5.87 (4.19 to 8.22)
**Children 14 years**							
Non-asthmatic mothers	Non-asthmatic fathers	18 558	807	4.40%	0%	1	1
Asthmatic mothers	Non-asthmatic fathers	1457	196	13.50%	9.10%	3.42 (2.88 to 4.06)	3.03 (2.46 to 3.73)
Non-asthmatic mothers	Asthmatic fathers	1756	159	9.10%	4.70%	2.19 (1.83 to 2.62)	1.95 (1.57 to 2.43)
Asthmatic mothers	Asthmatic fathers	156	22	14.10%	9.70%	3.61 (2.28 to 5.72)	3.48 (2.07 to 5.83)

Adjusted ORs have been adjusted for maternal age, maternal and paternal education, maternal and paternal smoking in pregnancy, maternal and paternal prepregnancy BMI and maternal and paternal atopy.

BMI, body mass index.

A similar pattern was observed at age 7 (table 1). The OR associated with maternal asthma was 3.45 (95% CI 3.04 to 3.92), while the OR associated with paternal asthma was 2.32 (95% CI 2.04 to 2.64) and the OR for children where both parents had asthma was 6.15 (95% CI 4.59 to 8.23).

At age 14, maternal asthma continued to be a stronger predictor of offspring asthma than paternal asthma (table 1). The OR was 3.42 (95% CI 2.88 to 4.06) for maternal asthma only, compared with 2.19 (95% CI 1.83 to 2.62) for paternal asthma only. However, the effect of both parents having asthma was slightly lower than at earlier ages (OR 3.61, 95% CI 2.28 to 5.72). The adjusted ORs showed similar trends.

### Offspring asthma and risk difference according to parental asthma status

At age 3, the absolute risk difference (RD) for asthma was 10.7% in children of asthmatic mothers only, compared with 5.3% in those with asthmatic fathers only (table 1). A similar pattern persisted at age 7, with RDs of 8.8% for maternal-only asthma and 5.0% for paternal-only asthma. By age 14, the maternal effect remained evident with RDs of 9.1% (maternal-only) versus 4.7% (paternal-only). At ages 3 and 7, the RD when both parents had asthma (16.8% and 16.7%, respectively) was approximately equal to the sum of the maternal and paternal effects. In contrast, by age 14, the RD for children with two asthmatic parents (9.7%) more closely resembled that of maternal-only asthma, suggesting a diminishing additive effect over time.

### Sex differences in the association between parental and offspring asthma

As expected, boys generally exhibited higher absolute risks of asthma than girls at ages 3 and 7, but the opposite was seen at age 14 (table 2). Overall, associations between parental and offspring asthma were similar across sexes. At age 3, adjusted ORs for maternal, paternal and both-parent asthma were comparable for boys and girls, with overlapping CIs and no statistically significant interactions observed (all interaction p values >0.24). At age 7, maternal asthma was associated with a higher OR for asthma in girls (OR 3.52, 95% CI 2.80–4.42) compared with boys (OR 2.63, 95% CI 2.15–3.23), and while CIs overlapped, the interaction between maternal asthma and sex approached statistical significance (p=0.065), suggesting possible sex-specific susceptibility. By age 14, adjusted ORs for parental asthma were again generally higher in girls than boys, particularly for both-parent asthma (girls: OR 4.38, 95% CI 2.28–8.43; boys: OR 2.60, 95% CI 1.11–6.10), though no interactions reached statistical significance (all p≥0.221). Overall, there was limited and inconsistent evidence of sex-specific modification in the association between parental and offspring asthma.

**Table 2:** Adjusted ORs calculated separately by offspring gender

Parental asthma status		Boys/girls n	Asthma Boys/girls n	Absolute risk Boys/girls	Risk difference Boys/girls	Adjusted OR for asthma	
						Boys	Girls
**Children 3 years**							
Non-asthmatic mothers	Non-asthmatic fathers	19 984/19 203	1239/807	6.2%/4.2%	0%/0%	1	1
Asthmatic mothers	Non-asthmatic fathers	1608/1452	308/177	19.2%/12.2%	13%/8%	3.30 (2.80–3.89)	2.82 (2.28–3.48)
Non-asthmatic mothers	Asthmatic fathers	1858/1847	237/152	12.8%/8.2%	6.6%/4%	2.25 (1.90–2.67)	2.28 (1.86–2.81)
Asthmatic mothers	Asthmatic fathers	175/175	47/30	26.9%/17.1%	20.7%/12.9%	4.56 (3.10–6.80)	3.82 (2.36–6.17)
**Children 7 years**							
Non-asthmatic mothers	Non-asthmatic fathers	18 222/17 363	891/573	4.9%/3.3%	0%/0%	1	1
Asthmatic mothers	Non-asthmatic fathers	1432/1327	208/148	14.5%/11.2%	9.6%/7.9%	2.63 (2.15–3.23)	3.52 (2.80–4.42)
Non-asthmatic mothers	Asthmatic fathers	1726/1657	175/131	10.1%/7.9%	5.2%/4.6%	2.18 (1.78–2.66)	2.64 (2.10–3.32)
Asthmatic mothers	Asthmatic fathers	152/146	38/24	25.0%/16.4%	20.1%/13.1%	7.21 (4.67–11.15)	4.50 (2.62–7.74)
**Children 14 years**							
Non-asthmatic mothers	Non-asthmatic fathers	9199/9359	413/394	4.5%/4.2%	0%/0%	1	1
Asthmatic mothers	Non-asthmatic fathers	752/705	100/96	13.3%/13.6%	8.8%/9.4%	2.93 (2.21–3.91)	3.16 (2.34–4.27)
Non-asthmatic mothers	Asthmatic fathers	883/873	72/87	8.2%/10.0%	3.7%/5.8%	1.70 (1.24–2.33)	2.28 (1.70–3.07)
Asthmatic mothers	Asthmatic fathers	77/79	12-Oct	12.1%/15.2%	7.6%/11%	2.60 (1.11–6.10)	4.38 (2.28–8.43)

### Differences by atopic versus non-atopic asthma

Child atopic trait data were available at ages 3 and 7, allowing stratified analyses by atopy status (online supplemental table 2). At age 3, both parental asthma and child atopy were independently associated with increased odds of asthma, but no significant interactions were observed (all interaction p values >0.17), suggesting limited evidence that atopy modified the association between parental and child asthma at this age. CIs were wide and overlapping, further limiting statistical certainty.

At age 7, a significant interaction was observed between paternal asthma and child atopy (interaction p=0.010), indicating that the association between paternal asthma and offspring asthma differed by atopy status. Specifically, the adjusted OR for paternal asthma was higher in non-atopic children (OR 2.44, 95% CI 2.07–2.89) than in atopic children (OR 1.44, 95% CI 0.96–2.17), suggesting a stronger relative association in non-atopic children. No significant interactions were found for maternal asthma (p=0.23) or when both parents were asthmatic (p=0.91), providing limited evidence of effect modification in those groups.

## Discussion

In this large population-based study, we found that maternal history of asthma is a stronger predictor for offspring asthma than paternal history, and that this effect is also present in adolescence. As expected, children of asthmatic parents had a higher prevalence of asthma at ages 3 years, 7 and 14 years compared with children of non-asthmatic parents. When examining asthma by offspring sex, we observed the well-established trend that boys are more affected in early childhood, whereas girls tend to have higher asthma prevalence later in childhood and adolescence.^22 23^ Despite these sex differences, the maternal effect on offspring asthma remained consistent across all age groups and in both sexes. We also did not find any notable differences in the relationship between parental and offspring asthma according to whether the asthma phenotype in the offspring was atopic or non-atopic.

We explored possible interactions between offspring sex, atopic traits and parental asthma status on childhood asthma, but found limited statistically significant interactions. Importantly, our findings persisted after adjusting for key parental background characteristics, including maternal age, maternal and paternal prepregnancy BMI, maternal and paternal education, maternal and paternal smoking in pregnancy and both maternal and paternal atopy. These results underscore the importance of maternal asthma history in predicting childhood asthma risk. Improving our understanding of these associations may enhance early identification and follow-up of children at increased risk of asthma.

Previous studies comparing the risk of childhood asthma associated with maternal versus paternal asthma have generally found that maternal asthma confers a higher risk, particularly in early childhood, up to 5 years of age.^11 14 24 25^ Consistent with this, we expected to observe a stronger maternal effect on early childhood asthma, as supported by several studies.^15 16^ In line with other researchers, Lim *et al*’s meta-analysis in 2010 concluded that maternal asthma increased the risk of offspring asthma more than paternal asthma (OR 3.04 vs 2.44, p=0.037).^14^ However, they did not find significant differences between maternal and paternal asthma when only looking at studies in which the patients were 5 years or older (OR 3.15 vs 2.60, p=0.14). Similarly, a US-based study from Harvard involving 499 families found that maternal asthma was a stronger predictor of asthma in children under 5 years (OR 5.0, 95% CI 1.7 to 14.9) compared with paternal asthma (OR 1.6, 95% CI 0.6 to 4.3), but after age 5, the risks associated with maternal and paternal asthma were similar (OR 4.6 vs 4.1).^11^

In contrast to these findings, our study demonstrates that the maternal effect persists beyond early childhood. Even at ages 7 and 14, maternal asthma was associated with a significantly higher risk of offspring asthma compared with paternal asthma. This suggests that maternal influences on asthma susceptibility may extend further into adolescence than previously thought. The maternal effect on asthma onset later in childhood, adolescence and even into adulthood remains controversial. A study conducted in Finland, including 477 cases and 842 controls, found that paternal asthma was associated with a greater risk of adult-onset asthma than maternal asthma (OR 2.52, 95% CI 1.66 to 3.83 vs OR 1.99, 95% CI 1.36 to 2.91). There is, however, a clear overlap in CIs in this study; hence, its statistical power is limited.^26^ In contrast to our findings is a cross-sectional survey of 9349, 9–11 years old schoolchildren in Munich and southern Bavaria that found that paternal asthma (OR 4.4, 95% CI 2.5 to 7.8) conferred a greater risk than maternal asthma (OR 1.5, 95% CI 0.7 to 2.7).^17^ However, more consistent with our findings, a cohort study from Denmark which included 15 014 children born to asthmatic mothers and 10 445 children born to asthmatic fathers found an increased prevalence of early-onset transient, early-onset persistent and late-onset asthma in the offspring of asthmatic mothers compared with those of asthmatic fathers.^27^ Variations in data collection methods and diagnostic criteria across studies may contribute to the differences in prevalence estimates.

Several theories have been proposed to explain the maternal effect on offspring asthma. One suggests that asthmatic mothers may have a different immune profile than non-asthmatic mothers,^12^ while another posits that immunological activity from airway inflammation in asthmatic mothers may contribute to epigenetic changes in the offspring.^28 29^ During pregnancy, the environment for the developing fetus is both affected by external environmental factors and the mother’s genetic makeup. The external factors, such as smoking, diet, medications and toxicants, all impact the intrauterine milieu, while the mother’s genetic predisposition also likely plays a role.^30^ A pregnant woman with asthma, particularly if her symptoms are active, may have a different immune profile compared with a non-allergic mother. For example, asthmatic mothers may secrete higher levels of T helper cell (Th)2 cytokines, which could influence foetal development and increase the risk of asthma later in life.^12^ Episodes of hypoxia in asthmatic mothers during pregnancy may have additional impact on the developing child.

Normally, a Th2-skewed immune response is promoted during pregnancy, but this response transitions to a non-allergic Th1 type after birth. However, little is known about whether this immune transition is delayed or prevented in allergic mothers. If it is delayed or prevented, it may predispose their children to allergic sensitisation or asthma.^31^ Studies in animal models have demonstrated that sensitising females during pregnancy can influence the phenotype of their offspring.^31^

A study conducted in Canada with 8226 children found that the incidence of asthma was higher in children born to women with moderate-to-severe uncontrolled asthma during pregnancy compared with those born to women with mild controlled asthma (adjusted OR 1.27, 95% CI 1.06 to 1.52).^32^ However, this study did not observe an increased risk of asthma in offspring of mothers with mild uncontrolled asthma or moderate-to-severe controlled asthma. These findings have been criticised for potential Berksonian bias.^33^

Complex epigenetic mechanisms are likely involved in the transmission of asthma from parents to offspring,^34^,^37^ with animal models suggesting that immunological activity, such as airway inflammation, may modify epigenetic characteristics.^28 29^ One study hypothesised that parental asthma severity, along with respiratory or allergic disease activity, could influence the offspring’s susceptibility to asthma, independent of genetic heritability.^38^ The study found that parental bronchial hyper-responsiveness and specific IgE levels, particularly before conception, were associated with an increased risk of offspring developing asthma and hay fever.

Maternal atopy and serum IgE levels have consistently been stronger predictors of cord-blood IgE levels than paternal atopy and IgE levels.^39 40^ A large Danish cohort study, which included parent-specific heritability of elevated specific IgE, demonstrated that maternal asthma history, elevated total IgE and allergic sensitisation posed a stronger risk for early childhood asthma and allergic traits than paternal history, especially in the years before the age of 6.^24^

### Strengths and limitations

Strengths of our study include the large sample size, which provides robust statistical power. However, several limitations should be noted. First, the broad exposure definition for parental asthma (at any point before or during pregnancy) may introduce heterogeneity and could attenuate effect estimates, as well as affect comparability with studies using more detailed clinical or temporal definitions. Nevertheless, parental asthma at any point in life is a widely used marker of familial and genetic susceptibility in studies of intergenerational asthma risk. Any misclassification of parental asthma is likely to be non-differential with respect to child asthma outcomes, which would generally bias associations toward the null and result in more conservative effect estimates.

Furthermore, maternal reports of asthma may lead to misclassification, potentially overestimating or underestimating true asthma prevalence. The mother’s understanding of asthma and her tendency to seek medical care might also influence how she responds to questionnaires. Mothers with asthma may be more likely to notice or report symptoms in their children, and although this could contribute to the stronger maternal–paternal associations observed, over-reporting is not necessarily restricted to mothers; fathers with asthma may also be particularly attentive to asthma symptoms in their children. Importantly, maternally reported asthma in MoBa has been validated against dispensed antiasthmatics in the NorPD, showing high specificity (96.8%) and good sensitivity (80–85%), with only 1.2% of children without reported asthma having dispensed antiasthmatics.^21^ These findings support the reliability of maternal report as a proxy for clinically meaningful asthma in large-scale epidemiological studies. However, because objective clinical data or prescription information for the offspring were not available for this study, we were unable to perform sensitivity analyses using stricter asthma definitions.

Moreover, although we adjusted for parental atopy to account for shared genetic predisposition, atopy may lie partly on the causal pathway between parental asthma and childhood asthma, introducing the possibility of overadjustment and attenuated associations.

Another limitation is potential selection bias due to the initial 41% participation rate in the MoBa cohort. Geographical differences in respiratory tract infection frequencies between rural and urban areas, as well as climate variations, could also impact asthma prevalence. While the results are consistent with those of other studies, the possibility remains that children and parents who participated in the cohort may have a higher health awareness or greater disease occurrence than the general population.

In addition, the analyses were performed in a cross-sectional manner, looking at three different points in time (ages 3, 7 and 14), rather than as a longitudinal follow-up. As a result, we did not investigate specific asthma phenotypes. While loss to follow-up was not a major challenge due to the cross-sectional approach, response rates were lower for the questionnaires completed by mothers of 14-year-olds, resulting in a smaller sample size for that age group compared with ages 3 and 7. Responders at age 14 were more likely to have highly educated parents, lower parental smoking rates and slightly older maternal age compared with the full MoBa cohort, although parental BMI distributions were similar. These differences may introduce some degree of selection bias. These patterns are consistent with previous methodological work in MoBa, which shows that while selective participation can affect prevalence estimates, exposure–outcome associations are generally only modestly influenced. Importantly, for attrition to bias our main conclusions, loss to follow-up would need to differ systematically between families with maternal versus paternal asthma. Online supplemental table 3 shows that attrition patterns were broadly similar across key sociodemographic factors, including parental education, suggesting that differential loss by parental asthma status is unlikely. Maternal asthma prevalence was also relatively stable across age groups (7.6% at age 3 vs 7.4% at age 14), supporting this interpretation. Therefore, any resulting bias is expected to be modest.^41^

Because some parents contributed multiple children to the cohort, we accounted for within-family clustering using robust standard errors. This reduces bias arising from correlated sibling data; however, results should still be interpreted at the family level, as exposure status is shared among siblings.

Finally, our study involved several comparisons across child ages and exploratory subgroup analyses (eg, by sex and parental atopy), increasing the potential for Type I error. Although we did not apply formal correction methods such as Bonferroni adjustment—given the biological motivation and non-independence of these analyses—we acknowledge that some subgroup findings should be interpreted with caution. Importantly, the primary pattern of stronger associations for maternal compared with paternal asthma was consistent across all ages, reducing the likelihood that our main conclusions reflect chance.

## Conclusion

Our findings support that maternal asthma contributes more significantly to offspring asthma than paternal asthma, with this effect persisting through childhood and into adolescence. These differences did not significantly vary according to offspring sex nor whether the child had additional atopic traits.

## Supplementary material

10.1136/bmjresp-2025-003608 online supplemental table 1

10.1136/bmjresp-2025-003608 online supplemental table 2

10.1136/bmjresp-2025-003608 online supplemental table 3

## Data Availability

Data are available upon reasonable request.
