# Supplementary material for: Parental asthma and risk of offspring asthma from childhood to adolescence: a population-based cohort study
Source: BMJ Open Respir Res. 2026 Jan 20;13(1):e003608. doi: 10.1136/bmjresp-2025-003608 (PMC12820850; doi:10.1136/bmjresp-2025-003608)
Supplement: online supplemental table 1 [file bmjresp-13-1-s001.docx]

|  | *Age 3: N= 46302* | | *Age 7: N= 42025* | | *Age 14:  N= 21927* | |  |
| --- | --- | --- | --- | --- | --- | --- | --- |
|  |  |  |  |  |  |  |  |
|  |  |  |  |  |  |  |  |
| **Characteristics** | ***Offspring asthma at age 3*** | | ***Offspring asthma at age 7*** | | ***Offspring asthma at age 14*** | |  |
|  | *n= 2997 (6.5%)* | | *n= 2188 (5.2%)* | | *n= 1184 (5.4%)* | |  |
|  |  | |  | |  | |  |
|  | **YES** | **NO** | **YES** | **NO** | **YES** | **NO** |  |
| **Ceaserian section** |  |  |  |  |  |  |  |
| Yes | 556 (8.3%) | 6181 (91.8%) | 438 (7.3%) | 5600 (92.8%) | 217 (7.0%) | 2864 (93.0%) |  |
| No | 2441 (6.2%) | 37124 (93.8%) | 1750 (4.9%) | 34237 (95.1%) | 967 (5.1%) | 17879 (94.9%) |  |
| **Maternal age at delivery** |  |  |  |  |  |  |  |
| **at delivery** |  |  |  |  |  |  |  |
| < 25 | 308 (7.6%) | 3748 (92.4%) | 221 (6.6%) | 3127 (93.4%) | 85 (5.6%) | 1426 (94.4%) |  |
| 25-29 | 1064 (6.9%) | 14450 (93.1%) | 752 (5.5%) | 12997 (94.5%) | 427 (6.2%) | 6482 (93.8%) |  |
| 30-34 | 1125 (6.0%) | 17489 (94.0%) | 816 (4.8%) | 16373 (95.3%) | 458 (4.9%) | 8812 (95.1%) |  |
| 35-39 | 446 (6.2%) | 6736 (93.8%) | 345 (5.0%) | 6496 (95.0%) | 192 (5.2%) | 3537 (94.9%) |  |
| 40+ | 54 (5.8%) | 882 (94.2%) | 54 (6.0%) | 844 (94.0%) | 22 (4.3%) | 486 (95.7%) |  |
| **Maternal BMI** |  |  |  |  |  |  |  |
| Underweight (BMI < 18.5) | 83 (6.5%) | 1196 (93.5%) | 71 (6.2%) | 1069 (93.8%) | 41 (6.8%) | 559 (93.2%) |  |
| Normal weight (BMI 18.5-24.9) | 1804 (6.0%) | 28441 (94.0%) | 1302 (4.7%) | 26382 (95.3%) | 739 (5.1%) | 13838 (94.9%) |  |
| Overweight (BMI 25.0-29.9) | 684 (7.0%) | 9078 (93.0%) | 506 (5.7%) | 8305 (94.3%) | 240 (5.2%) | 4336 (94.8%) |  |
| Obese (>30) | 328 (8.7%) | 3442 (91.3%) | 247 (8.0%) | 3007 (92.4%) | 123 (7.5%) | 1509 (92.5%) |  |
| Missing | 98 (7.9%) | 1148 (92.1%) | 62 (5.5%) | 1074 (94.5%) | 41 (7.6%) | 501 (92.4%) |  |
| **Paternal BMI** |  |  |  |  |  |  |  |
| Underweight (BMI < 18.5) | 8 (7.9%) | 93 (92.1%) | 10 (11.1%) | 80 (89.9%) | 2 (4.4%) | 43 (95.6%) |  |
| Normal weight (BMI 18.5-24.9) | 1228 (6.1%) | 18922 (93.9%) | 884 (4.8%) | 17604 (95.2%) | 494 (5.1%) | 9224 (94.9%) |  |
| Overweight (BMI 25.0-29.9) | 1302 (6.4%) | 19182 (93.6%) | 959 (5.2%) | 17636 (94.8%) | 524 (5.4%) | 9125 (94.6%) |  |
| Obese (>30) | 377 (8.4%) | 4117 (91.6%) | 289 (7.4%) | 3626 (23.6%) | 130 (6.6%) | 1854 (93.5%) |  |
| Missing | 82 (7.6%) | 991 (92.4%) | 46 (4.9%) | 891 (95.1%) | 34 (6.4%) | 497 (93.6%) |  |
| **Maternal smoking** |  |  |  |  |  |  |  |
| At start of pregnancy |  |  |  |  |  |  |  |
| Yes | 805 (7.2%) | 10409 (92.8%) | 537 (5.5%) | 9279 (94.5%) | 265 (5.4%) | 4626 (94.6%) |  |
| No | 1925 (6.4%) | 28377 (93.7%) | 1411 (5.1%) | 26484 (94.9%) | 808 (5.4%) | 14240 (94.6%) |  |
| Missing | 267 (5.6%) | 4519 (94.4%) | 240 (5.6%) | 4074 (94.4%) | 111 (5.6%) | 1877 (94.4%) |  |
| Smoking at week 15 of pregnancy |  |  |  |  |  |  |  |
| Yes | 220 (8.0%) | 2522 (92.0%) | 141 (6.4%) | 2062 (93.6%) | 64 (6.1%) | 989 (93.9%) |  |
| No | 2531 (6.5%) | 36667 (93.5%) | 1834 (5.1%) | 34060 (94.9%) | 1018 (5.3%) | 18064 (94.7%) |  |
| Missing | 246 (5.6%) | 4116 (94.4%) | 213 (5.4%) | 3715 (94.6%) | 102 (5.7%) | 1690 (94.3%) |  |
| Smoking at child age 3 yrs |  |  |  |  |  |  |  |
| Yes | 431 (7.5%) | 5289 (92.5%) | 217 (5.5%) | 3712 (94.5%) | 103 (5.4%) | 1824 (94.7%) |  |
| No | 2477 (6.4%) | 36279 (93.6%) | 1505 (5.2%) | 27738 (94.9%) | 810 (5.2%) | 14689 (94.8%) |  |
| Missing | 89 (4.9%) | 1737 (95.1%) | 466 (5.3%) | 8387 (94.7%) | 271 (6.0%) | 4230 (94.0%) |  |
| **Paternal smoking** |  |  |  |  |  |  |  |
| Smoking at week 15 of pregnancy |  |  |  |  |  |  |  |
| Yes | 543 (7.1%) | 7094 (92.9%) | 391 (6.0%) | 6183 (94.1%) | 193 (5.9%) | 3100 (94.1%) |  |
| No | 2415 (6.3%) | 35687 (93.7%) | 1772 (5.1%) | 33190 (94.9%) | 976 (5.3%) | 17411 (94.7%) |  |
| Missing | 39 (6.9%) | 524 (93.1%) | 25 (5.1%) | 464 (94.9%) | 15 (6.1%) | 232 (93.9%) |  |
| **Maternal education status** |  |  |  |  |  |  |  |
| 9-yr elementary school | 55 (7.8%) | 650 (92.2%) | 45 (8.0%) | 518 (92.0%) | 23 (10.6%) | 194 (89.4%) |  |
| 1-3 yrs of high school | 903 (7.4%) | 11285 (92.6%) | 672 (6.3%) | 9932 (93.7%) | 285 (5.9%) | 4551 (94.1%) |  |
| 4 + yrs of university /college | 1886 (6.0%) | 29369 (94.0%) | 1370 (4.7%) | 27518 (95.3%) | 814 (5.1%) | 15044 (94.9%) |  |
| Missing | 153 (7.1%) | 2001 (92.9%) | 101 (5.1%) | 1869 (94.9%) | 62 (6.1%) | 954 (93.9%) |  |
| **Paternal education status** |  |  |  |  |  |  |  |
| 9-yr elementary school | 108 (7.4%) | 1347 (92.6%) | 81 (6.5%) | 1165 (93.5%) | 35 (6.3%) | 525 (93.8%) |  |
| 1-3 yrs of high school | 1229 (7.2%) | 15950 (92.9%) | 920 (6.1%) | 14217 (93.9%) | 425 (5.7%) | 6988 (94.3%) |  |
| 4 + yrs of university /college | 1423 (6.0%) | 22433 (94.0%) | 995 (4.5%) | 21212 (95.5%) | 602 (5.0%) | 11567 (95.1%) |  |
| Missing | 237 (6.2%) | 3575 (93.8%) | 192 (5.6%) | 3243 (94.4%) | 122 (6.8%) | 1663 (93.2%) |  |
| **Maternal atopy only** |  |  |  |  |  |  |  |
| Yes | 778 (8.7%) | 8130 (91.3%) | 571 (7.1%) | 7503 (92.9%) | 285 (6.6%) | 4054 (93.4%) |  |
| No | 2219 (5.9%) | 35175 (94.1%) | 1617 (4.8%) | 32334 (95.2%) | 899 (5.1%) | 16689 (94.9%) |  |
| **Paternal atopy only** |  |  |  |  |  |  |  |
| Yes | 546 (7.1%) | 7162 (92.9%) | 407 (5.8%) | 6650 (94.2%) | 159 (9.1%) | 1597 (91.0%) |  |
| No | 2451 (6.4%) | 36143 (93.7%) | 1781 (5.1%) | 33187 (94.1%) | 1025 (5.1%) | 19146 (94.9%) |  |
| **Sex of the child** |  |  |  |  |  |  |  |
| Boy | 1831 (7.8%) | 21794 (92.3%) | 1312 (6.1%) | 20220 (93.9%) | 595 (5.5%) | 10316 (94.6%) |  |
| Girl | 1166 (5.1%) | 21511 (94.9%) | 876 (4.3%) | 19617 (95.7%) | 589 (5.4%) | 10427 (94.7%) |  |
| **Birthweight** |  |  |  |  |  |  |  |
| 250g-999g | 21 (24.4%) | 65 (75.6%) | 19 (24.1%) | 60 (76.0%) | 9 (16.7%) | 45 (83.3%) |  |
| 1000g-1499g | 35 (19.0%) | 149 (81.0%) | 21 (12.9%) | 142 (87.1%) | 7 (8.5%) | 75 (91.5%) |  |
| 1500g-2499g | 162 (10.5%) | 1382 (89.5%) | 113 (8.1%) | 1287 (91.9%) | 41 (5.7%) | 673 (94.3%) |  |
| 2500g-4000g | 2172 (6.2%) | 32838 (93.8%) | 1561 (4.9%) | 30242 (95.1%) | 882 (5.4%) | 15550 (94.6%) |  |
| >4000g | 607 (6.4%) | 8871 (93.6%) | 474 (5.5%) | 8106 (94.5%) | 245 (5.3%) | 4400 (94.7%) |  |

**Supplemental table 1:** Distribution of maternal, paternal and offspring characteristics by offspring asthma status at age 3, 7 and 14.
