# Supplementary material for: Parental asthma and risk of offspring asthma from childhood to adolescence: a population-based cohort study
Source: BMJ Open Respir Res. 2026 Jan 20;13(1):e003608. doi: 10.1136/bmjresp-2025-003608 (PMC12820850; doi:10.1136/bmjresp-2025-003608)
Supplement: online supplemental table 2 [file bmjresp-13-1-s002.docx]

|  |  | **Children 3 years** | | | | | |  |
| --- | --- | --- | --- | --- | --- | --- | --- | --- |
| **Parental asthma status** | | **No Atopy/ Atopy n=** | **Asthma No Atopy/ Atopy n=** | **Absolute risk No Atopy/ Atopy** | **Risk difference No Atopy/ Atopy** | **Adjusted OR for asthma No Atopy / Atopy** | |  |
|  |  |  |  |  |  |  |  |  |
| **Non-asthmatic  mothers** | **Non-asthmatic  fathers** | 38862/325 | 1942/ 104 | 5.0%/32.0% | 0%/0% | **1** | **1** |  |
|  |  |  |  |  |  |  |  |  |
| **Asthmatic  mothers** | **Non-asthmatic  fathers** | 2968/92 | 436/ 49 | 14.7%/53.3% | 9.7%/21.3% | **2.99 (2.61-3.42)** | **2.62 (1.47-4.69)** |  |
|  |  |  |  |  |  |  |  |  |
| **Non-asthmatic  mothers** | **Asthmatic  fathers** | 3643/62 | 361/ 28 | 9.9%/45.2% | 4.9%/13.2% | **2.22 (1.94-2.54)** | **1.95 (0.99-3.82)** |  |
|  |  |  |  |  |  |  |  |  |
| **Asthmatic  mothers** | **Asthmatic  fathers** | 334/16 | 69/ 8 | 20.7%/50.0% | 15.7%/18.0% | **4.02 (2.90-5.59)** | **2.33 (0.64-8.42)** |  |
|  |  |  |  |  |  |  |  |  |
|  |  | **Children 7 years** | | | | | |  |
| **Parental asthma status** | | **No Atopy/ Atopy n=** | **Asthma No Atopy/ Atopy n=** | **Absolute risk No Atopy/ Atopy** | **Risk difference No Atopy/ Atopy** | **Adjusted OR for asthma No Atopy / Atopy** | |  |
|  |  |  |  |  |  |  |  |  |
| **Non-asthmatic  mothers** | **Non-asthmatic  fathers** | 28135/1074 | 1210/ 254 | 3.5%/19.4% | 0%/0% | **1** | **1** |  |
|  |  |  |  |  |  |  |  |  |
| **Asthmatic  mothers** | **Non-asthmatic  fathers** | 2077/179 | 277/ 79 | 10.9%/36.6% | 7.4%/17.2% | **2.89 (2.44-3.43)** | **2.23 (1.55-3.20)** |  |
|  |  |  |  |  |  |  |  |  |
| **Non-asthmatic  mothers** | **Asthmatic  fathers** | 2593/156 | 258/ 48 | 8.1%/24.7% | 4.6%/5.3% | **2.44 (2.07-2.89)** | **1.44 (0.96-2.17)** |  |
|  |  |  |  |  |  |  |  |  |
| **Asthmatic  mothers** | **Asthmatic  fathers** | 208/33 | 40/ 22 | 15.6%/52.4% | 12.1%/33% | **4.53 (3.02-6.79)** | **4.99 (2.51-9.91)** |  |
|  |  |  |  |  |  |  |  |  |

**Supplemental table 2:** Adjusted odds ratios, clustered at maternal level, calculated separately by offspring atopy status.
