# Supplementary material for: Parental asthma and risk of offspring asthma from childhood to adolescence: a population-based cohort study
Source: BMJ Open Respir Res. 2026 Jan 20;13(1):e003608. doi: 10.1136/bmjresp-2025-003608 (PMC12820850; doi:10.1136/bmjresp-2025-003608)
Supplement: online supplemental table 3 [file bmjresp-13-1-s003.docx]

|  | **MoBa cohort**  *n = 102862* | **Age 3**  *n=46302* | **Age 7**  *n=42025* | **Age 14**  *n=21927* |
| --- | --- | --- | --- | --- |
| **Characteristics** |  |  |  |  |
|  |  |  |  |  |
| **Maternal asthma** |  |  |  |  |
| Yes | 7761 (7.6%) | 3410 (7.4%) | 3057 (7.3%) | 1613 (7.4%) |
| No | 95101 (92.5%) | 42892 (92.6%) | 38968 (92.7%) | 20314 (92.6%) |
| **Maternal age at delivery** |  |  |  |  |
| < 25 | 11206 (10.9%) | 4056 (8.8%) | 3348 (8.0%) | 1511 (6.9%) |
| 25-29 | 33704 (32.8%) | 15514 (33.5%) | 13749 (32.7%) | 6909 (31.5%) |
| 30-34 | 39855 (38.8%) | 18614 (40.2%) | 17189 (41.0%) | 9270 (42.3%) |
| 35-39 | 15970 (15.5%) | 7182 (15.5%) | 6841 (16.3%) | 3729 (17.0%) |
| 40+ | 2127 (2.1%) | 936 (2.0%) | 898 (2.1%) | 508 (2.3%) |
| **Maternal BMI** |  |  |  |  |
| Underweight (BMI < 18.5) | 3070 (3.0%) | 1279 (2.8%) | 1140 (2.7%) | 600 (2.7%) |
| Normal weight (BMI 18.5-24.9) | 65433 (63.6%) | 30245 (65.3%) | 27684 (65.9%) | 14577 (66.5%) |
| Overweight (BMI 25.0-29.9) | 21870 (21.3%) | 9762 (21.1%) | 8811 (21.0%) | 4576 (20.1%) |
| Obese (>30) | 9013 (8.8%) | 3770 (8.1%) | 3254 (7.7%) | 1632 (7.4%) |
| Missing | 3476 (3.4%) | 1246 (2.7%) | 1136 (2.7%) | 542 (2.5%) |
| **Paternal BMI** |  |  |  |  |
| Underweight (BMI < 18.5) | 267 (0.3%) | 101 (0.2%) | 90 (0.2%) | 45 (0.2%) |
| Normal weight (BMI 18.5-24.9) | 43951 (42.7%) | 20150 (43.5%) | 18488 (44.0%) | 9718 (44.3%) |
| Overweight  (BMI 25.0-29.9) | 44964 (43.7%) | 20484 (44.2%) | 18595 (44.3%) | 9649 (44.0%) |
| Obese (>30) | 10038 (9.8%) | 4494 (9.7%) | 3915 (9.3%) | 1984 (9.1%) |
| Missing | 3642 (3.5%) | 1073 (2.3%) | 937 (2.2%) | 531 (2.4%) |
| **Maternal smoking** |  |  |  |  |
| Smoking at week 15 of pregnancy |  |  |  |  |
| Yes | 8523 (8.3%) | 2742 (5.9%) | 2203 (5.2%) | 1053 (4.8%) |
| No | 82571 (80.3%) | 39198 (84.7%) | 35894 (85.4%) | 19082 (87.0%) |
| Missing | 11768 (11.4%) | 4362 (9.4%) | 3928 (9.4%) | 1792 (8.2%) |
| **Paternal smoking** |  |  |  |  |
| Smoking at week 15 of pregnancy |  |  |  |  |
| Yes | 20863 (20.3%) | 7637 (16.5%) | 6574 (15.6%) | 3293 (15.0%) |
| No | 80452 (78.2%) | 38102 (82.3%) | 34962 (83.2%) | 18387 (83.9%) |
| Missing | 1547 (1.5%) | 563 (1.2%) | 489 (1.2%) | 247 (1.1%) |
| **Maternal education status** |  |  |  |  |
| 9-yr elementary school | 2765 (2.7%) | 705 (1.5%) | 563 (1.3%) | 217 (1.0%) |
| 1-3 yrs of high school | 32135 (31.2%) | 12188 (26.3%) | 10604 (25.2%) | 4836 (22.1%) |
| 4 + yrs of university /college | 62722 (61.0%) | 31255 (67.5%) | 28888 (68.7%) | 15858 (72.3%) |
| Missing | 5240 (5.1%) | 2154 (4.7%) | 1970 (4.7%) | 1016 (4.6%) |
| **Paternal education status** |  |  |  |  |
| 9-yr elementary school | 4644 (4.5%) | 1455 (3.1%) | 1246 (3.0%) | 560 (2.6%) |
| 1-3 yrs of high school | 41435 (40.3%) | 17179 (37.1%) | 15137 (36.0%) | 7413 (33.8%) |
| 4 + yrs of university /college | 47429 (46.1%) | 23856 (51.5%) | 22207 (52.8%) | 12169 (55.5%) |
| Missing | 9334 (9.1%) | 3812 (8.2%) | 3435 (8.2%) | 1785 (8.1%) |

**Supplemental Table 3:** Comparison of baseline characteristics between the total MoBa cohort, and the responders at ages 3, 7 and 14 years.
